# A Resume of the Treatment of Gonorrhea, with Report of Cases

**Published:** 1904-02

**Authors:** Clarence G. Clark

**Affiliations:** New York; Assistant Surgeon, New York Hospital of Relief, Genitourinary Clinic; formerly assistant Surgeon, New York Hospital O. P. D., Genitourinary Department.


### A Resume of the Treatment of Gonorrhea, with Report of Cases.

By CLARENCE G. CLARK, M. D., New York.

Assistant Surgeon, New York Hospital of Relief, Genitourinary Clinic; formerly assistant Surgeon, New York Hospital O. P. D., Genitourinary Department.

GONORRHEA may be divided into two main groups—namely, acute and chronic, according to duration; and into two classes—anterior and posterior—according to the location of the disease. In the beginning all cases are anterior, that is, confined to the anterior or penile portion of the urethra, but from inefficient treatment or neglect many become posterior by direct extension. Between the two stages, acute and chronic, we have in many instances an intermediary stage, or so-called subacute. This stage is accompanied by very little pain and by a thin watery discharge, which is very often entirely free from gonococci. As the disease progresses backward another acute stage is very often developed, when the posterior urethra becomes involved. This is accompanied by severe pain which is often referred to the rectum. The discharge in this stage may be very slight or entirely absent, and is often thin and watery.

As chronicity becomes more marked, the pain decreases, and the discharge dwindles to the morning drop. This stage of the disease is the most obstinate and requires months of careful treatment before satisfactory results are obtained. It is generally complicated by strictures requiring the use of sounds and other means of dilatation. The seat of the process in this stage is generally the posterior urethra, although the anterior may be involved. This, however, I think, is rare. A chronic posterior urethritis may last for years, and while it causes very slight inconvenience to the patient, it is a constant source of danger to others. Such a patient lulled into a false sense of security by the very mildness of his symptoms may marry and then cause untold suffering to an innocent woman. It should be every physician's duty, whether genitourinary surgeon or a general practitioner, to check the disease before it reaches this stage, and this, I think, is possible in all cases if seen early enough. It has been my fortune, through my connection with a large metropolitan dispensary, to observe gonorrhea in all its sttages, and it is for the purpose of refreshing the practitioner's mind and of reviewing some of the newer methods of treatment that this paper has been written.

Of late years the profession has been so flooded with preparations possessed of absolutely no value, and many of which are merely old remedies under new and high-sounding names, that one of necessity looks with scepticism upon everything new brought before him. In no field is this so true as in genitourinary surgery where the disease under discussion offers such varied symptoms, for the relief of which new remedies are constantly introduced. It has, therefore, been my custom whenever anything new appealed to me particularly to test it carefully in a number of selected cases.

In the treatment of gonorrhea we have at our disposal three distinct methods in the beginning of the disease. These may be enumerated as follows: (1) symptomatic, *i. e.,* by internal medication designed to relieve special symptoms as they manifest themselves; (2) by hand injection; (3) by irrigation. The last named came into considerable prominence of late years and for a time promised much, but recently it has gone into more or less disuse. Many of the leading genitourinary surgeons have found that they can get better results with less danger of extending the infection by hand injections, since the force can be better regulated with these than with the irrigator, and although the quantity of solution used in the former method is less, the strength can be advantageously increased. Probably the sole advantage of the irrigator over other methods is the impression made upon the patient.

The best method of treating acute and subacute gonorrhea is then a combination of the first two methods, *i. e.,* symptomatic and the use of hand injections. The injections are best made by the physician himself and at least once daily. The patient is apt to use too great force in injecting himself. In the symptomatic or internal treatment we have in acute cases one marked symptom to relieve, *i. e.,* the pain on urination. Our effort should be to relieve this and at the same time to render the urine antiseptic. For this purpose I have derived excellent results from a comparatively new urinary antiseptic, a combination of anhydromethylen citric acid with hexamethylen-tetramin. The latter substance is an old remedy, and has been extensively used. Its action depends upon the liberation of formaldehyde, the amount set free, however, being under certain circumstances very small, especially if the urine is markedly alkaline, and in strongly ammoniacal urine it is practically *nil.* But in combination with anhydromethylen citric acid its efficiency is augmented, the formaldehyde being readily liberated both in alkaline and acid urine. Aside from its sterilising the urine it renders it less irritating, and thereby relieves the pain during micturition.

I have recently used this preparation, which is known as helmitol, in several cases of acute and chronic urethritis with excellent results. Nearly all the patients experienced relief immediately (within 24 hours) after taking it. Helmitol possesses an advantage over urotropin (ammonium formaldehyde) in being well tolerated even in large doses. It has proved especially useful in relieving the tenesmus of posterior urethritis and prostatitis, and I have not found it to be contraindicated in acute gonorrhea, being used in connection with hand injections. In three cases where the pain on urinating was intense it afforded relief in 24 hours.

I will describe below in detail six cases illustrating my present method of treatment, each representing a different stage of gonorrhea. Three of them I observed at the dispensary; the other three, including a case of cystitis, occurred in private practice.

Case I.—M. B., printer, 18 years of age, contracted gonorrhea eight days ago. First noticed burning on urination three days ago, which rapidly became worse and was accompanied by a thick yellow discharge. He complained that the pain on urinating was excruciating and that he voided his urine about every 15 minutes. He had very little sleep for the past two nights; discharge was profuse. Examination: discharge reveals large numbers of gonococci; urine acid in reaction and very high colored; inguinal glands enlarged on both sides. Treatment: helmitol, 15 grains every four hours. Injections of protargol, 1 per cent. were used, but the pain was so severe that they had to be discontinued. Instructed the patient to call daily. On the next visit he stated that the pain was much relieved, and that he voided larger quantities of urine at less frequent intervals. On the second day the pain was entirely gone. I then decreased the helmitol to 15 grains twice daily, which was sufficient to control the pain. I also resumed the use of injections of protargol 1 per cent., injecting two syringefuls daily (each containing 2 drachms) until the eighth day, when the discharge was very watery in character, and on examination contained very few gonococci. I then substituted zinc sulphocarbolate (2 grains to the ounce), which I injected for three more days, at which time the discharge had ceased entirely. On the twelfth day after beginning treatment this patient had absolutely no discharge and successfully passed the beer test.

Case II.—J. H., aged 26 years, carpenter, has had gonorrhea for four weeks. The pain of the acute stage diminished at the end of the first week, and the patient who had been using advertised preparations thought himself cured, although he noticed a slight discharge every morning. Has had six previous attacks. He first came to my office on November 3, 1903, complaining of severe pain in the rectum; discharge very thin and watery; frequent urination; urine cloudy; reaction, alkaline; pain is increased immediately after urinating and is especially severe at night, keeping him awake. Microscopical examination reveals gonococci in the discharge. Diagnosis: acute posterior urethritis, following an anterior urethritis of specific origin. On examination per rectum the prostate was found enlarged and tender. Treatment: the first thing to be done was manifestedly to relieve the tenesmus. For this I administered helmitol in 15-grain doses every four hours, and gave no other treatment at first. On November 4, the pain was no better, so the helmitol was increased to 20-grain doses every four hours. On November 5, the pain was less severe, the patient having had a good night. The drug was continued in the same dose, and on the sixth the pain was nearly gone. On this day the patient showed me a rash on his arm. This was papular in character and extended over the extensor surface of the right forearm. Whether due to helmitol or not I am unable to state, as I have never observed the same rash following the use of the drug. On November 6, I discontinued the helmitol, as the pain was gone, and began washing out the bladder with a solution of potassium permanganate, 1-3000, and massaging the prostate. This patient is still under treatment, as the discharge has not entirely disappeared, and the case is only cited to show the action of helmitol in relieving the tenesmus of acute posterior gonorrhea. I am now passing sounds twice weekly, and giving him deep injections of a 2 per cent. solution of protargol. The prostate has decreased in size, and he has not had any recurrence of pain on urinating nor of the tenesmus.

Case III.—A. G., aged 23 years, machinist; no previous history; intercourse five days ago; first noticed burning on urination last night which increased, until today it is very severe. Discharge first noticed this morning, it being very thick and yellowish. Examination under microscope reveals gonococci in large numbers. Pain is so severe that I did not consider it wise to inject or irrigate. Wishing to try the effect of helmitol in relieving the ardor urinæ accompanying the early stages of gonorrhea I perscribed it in 15-grain doses every four hours. Second day: pain no better; discharge worse. Last night patient suffered considerably from painful erections. I increased the helmitol to 18 grains, every four hours, and prescribed lupulin gr. 2, and camphor monobromate gr. 1, in pills to be taken every three hours during the evening. Third day; pain diminished, but still severe during urination; chordee did not trouble him much last night; continued the same treatment. Fourth day: pain completely gone. Discharge is, however, worse and I started irrigation with protargol 1-500; still continued helmitol. Fifth day: pain gone; discontinued helmitol; continued irrigation daily, increasing the protargol to 1-300 by the ninth day; discharge gradually diminished till by the twelfth day there was only a thick watery secretion, which contained no gonococci. I stopped the irrigations on this day and injected zinc sulphocarbolate, 4 grains to the ounce, for three days longer, by which time the discharge was all gone and treatment was discontinued. Patient has had no recurrence. In this case this formaldehyde compound certainly relieved the pain of the early stages of an acute gonorrhea, and I could see no reason why it is contraindicated in this stage.

Case IV., W. F., aged 21 years, first visit November 4, 1903, has had gonorrhea for eight weeks; has had more or less pain on urinating right along with discharge, which from a thick, creamy consistency has become more or less watery. About four days ago he was seized with severe urinary tenesmus with pain on micturition and slight hematuria. This has been getting worse until last night he had to get up twelve times to pass urine, which was streaked with blood and caused him great pain while passing it. No gonococci could be found in the sediment; no albumin. I prescribed helmitol, 15 grains every four hours, and injected the posterior urethra and bladder with warm bicarbonate of sodium solution. On November 5 the urine was still turbid and pain remained the same. On testing the urine with phloroglucin and sodium hydrate for formaldehyde a strong reaction occurred. Treatment continued. November 6, patient slightly better; passed urine only seven times during the night. Urine is no longer blood-stained, and the first portion is clearer than the second. Dose of helmitol raised to 20 grains, every four hours. November 7, the patient much easier; passes larger quantities of urine and much less frequently; both portions are clear; tenesmus is markedly diminished. Treatment continued, with the exception that I substituted a 1-500 solution of protargol for the bicarbonate in the irrigator. November 8, pain and tenesmus practically gone; only passed urine twice during the night; continued the helmitol for two days more, when the pain was completely gone; the discharge remained about the same. On passing a sound, I found stricture in the posterior urethra which would only admit the introduction of a 22 F sound. I began dilating this and injecting 2 per cent. protargol solution through a Keyes-Ultzmann deep syringe. This patient is still under treatment, and has improved so that a sound is passed only twice a week. His discharge is almost gone, and I can pass a 30 F sound without difficulty.

Case V.—W. B., aged 68 years; gonorrhea 30 years ago, from which he developed a stricture. This has not troubled him much in past ten years, but for the last month he has been troubled with frequent urination accompanied by tenesmus; this has been getting worse until he is now urinating every 10 to 15 minutes, the desire not leaving him when urine is voided. On examination I found that the stricture was so tight that it was with difficulty that even a No. 14 F could be passed. His prostate was also enlarged.

The urine was strongly ammoniacal from decomposition; tenderness over pubes. Diagnosis: acute cystitis resulting from retention of urine caused by stricture and prostatic hypertrophy. No gonococci were found in the urinary sediment and there was no discharge. I prescribed helmitol 15 grains three times daily; prostatic massage and dilatation of stricture. The patient was put to bed and upon a strict milk diet. The helmitol was given in large amounts of water. For the first three days a catheter was inserted to drain the bladder, washing it out with a solution of bicarbonate of sodium every day. The pain and tenesmus rapidly disappeared and the urine cleared. By the third day I was able to remove the catheter and begin the passage of sounds and bladder irrigation daily. The helmitol was continued for a week, by which time he voided urine naturally and only a trifle more frequently than normal. In two weeks the stricture was dilated sufficiently to allow the passage of a No. 30 F sound. For the bladder washing I substituted protargol in 1-300 for the bicarbonate of sodium. This case was discharged at the end of a month not as a permanent cure, but temporary. It is impossible to permanently cure these cystitis cases caused by prostatitis in the aged. The case, however, serves a good example of the action of helmitol in preventing fermentation, and thus relieving the tenesmus.

Case VI.—J. J., aged 20 years, has had gonorrhea for five days; thick discharge containing large numbers of gonococci; ardor urinæ is not very severe, but he has no control over the bladder muscles, micturating almost without warning. Urine is alkaline in reaction. Treatment: I prescribed helmitol in 15 grain doses after meals, with 1-60 grain of strychnia before meals; injected the patient daily with 1 per cent. protargol solution. The frequency of urination lessened after two days of this treatment and pain entirely disappeared. Continued the injections daily. In giving these a two-dram syringe is used, and the anterior urethra is filled with the solution which is allowed to remain for about five minutes by squeezing the lips of the meatus with the fingers. This is repeated once and should be done daily or twice daily, if possible. The discharge in this case diminished by the eighth day, when it became thin and watery. I then substituted zinc sulphocarbolate, 4 grs. to the ounce, for the protargol, and by the twelfth day the patient was discharged cured, having successfully passed the beer test. The helmitol in this case was continued throughout and succeeded admirably in relieving the irritation at the bladder neck.

These six cases represent various phases of uncomplicated gonorrhea and, I think, suffice to show the treatment used. In some cases it will be noted the irrigation treatment was employed, but in the average acute cases of gonorrhea I prefer hand injections. In the posterior cases where the process has already traveled as far back as it will go, the irrigation method will cover more ground and reach more of the diseased membrane than will the injections, and in those cases I have found it preferable.

I have employed helmitol with distinct service in almost all cases of gonorrhea in rendering the urine bland and antiseptic. In warding off the urethral chills, so often experienced after passing sounds or after operations on the urinary tract, I have found it to be almost a specific. I made it a custom to administer helmitol in 15-grain doses, three times daily, to all patients requiring frequent passage of sounds, and since its use have had no cases of urethral chill. It is preferable to ammonium formaldehyde in absence of irritating qualities and in not interfering with the digestion in the least.

As to the preparation used for injecting protargol, but little need be said, as it is so well known already. It has proven itself to be of more service in gonorrheal inflammations than the older antiseptics, such as bichloride of mercury, potassium permanganate, and the like. It can be used as an injection in ½ to 2 per cent. solutions or as an irrigation in 1-300 to 1-500 solution. In posterior urethritis it is my custom to irrigate the posterior urethra and bladder and to give the patient deep instillations through a Keyes-Ultzmann syringe. These cases almost invariably require the passage of sounds as they nearly all have some degree of stricture.

For the complications of gonorrhea but little can be said here, as they are too numerous to more than mention them in an article of this character. For epididymitis and orchitis I have found guaiacol and olive oil, equal parts, to be very efficient. This should be applied lightly to the scrotum every night with a camel's hair brush. A suspensory bandage should be worn, or the testicles may be strapped with adhesive plaster. These are the more frequently met with of the serious complications of gonorrhea, and it is hardly necessary to mention the others. If the acute case is seen early enough and handled properly the complications will be comparatively few.

335 East Eighty-seventh Street.

---

ANTI-RHEUMATIC.

℞ Sol. et pot. tartrat.................................. ½ oz.

Tongaline, q. s. ad.................................. 3 ozs.

M. Sig.—A teaspoonful three times a day.

